# Unusual Tonsillar Herniation in Meningeal Melanocytoma: A Case Report

**DOI:** 10.5812/iranjradiol.8766

**Published:** 2012-11-20

**Authors:** Kaveh Samimi, Mohammad Hadi Gharib, Kiara Rezaei-Kalantari, Maryam Jafari

**Affiliations:** 1 Department of Radiology, Rasoul-e-Akram Hospital, Tehran University of Medical Sciences, Tehran, Iran

**Keywords:** Tonsillar Herniation, Meningeal Neoplasms, Melanocyte

## Abstract

Meningeal melanocytoma is a primary melanocytic neoplasm with certain MR and immunohistochemical characteristics worthy to note. In a 38-year-old man with a complaint of headache for a couple of years and recently added nausea, vomiting, diplopia, progressive visual blurring and hearing loss, magnetic resonance imaging (MRI) was remarkable for T1 shortening of leptomeninges and certain nodules in precontrast study. Subsequent contrast-enhanced MR imaging of the brain and spine revealed enhancement in the basal cisterns extending throughout the spinal canal. Contrast-enhanced MRI revealed diffuse enhancement in the basal cisterns extending throughout the spinal canal. Immunohistochemical analysis on one of the intraspinal nodules proposed leptomeningeal melanocytoma. The characteristic shortening of T1 and T2 relaxation times in MRI as a result of the paramagnetic stable free radicals that exist within melanin, often suggests a diagnosis of a melanocytic leptomeningeal process. Moreover, there are unique immunohistochemical characteristics for these varied lesions. In appropriate clinical settings, certain radiologic findings, especially both T1 and T2 shortening in nodular CNS lesions should propose meningeal melanocytoma.

## 1. Introduction

Meningeal melanocytoma, as a primary melanocytic neoplasm, can exhibit distinguishing radiologic characteristics. This is useful, since a good postoperative survival rate is expected for patients with meningeal melanocytoma if complete resection is undertaken. Hence, the surgeon should be advised of this possible diagnosis in suspected cases of meningioma. Notions that can make the surgeon accomplish maximal effort at tumor resection and eliminate the risk of recurrence (1, 2).

## 2. Case Presentation

A 38-year-old man with a complaint of headache for a couple of years and recently added nausea, vomiting, diplopia, progressive visual blurring and hearing loss came to the hospital four years ago. Neurological examinations disclosed the following findings; visual acuity diminution, bilateral papillary edema, sensory-neural hearing loss, mild gait ataxia, bilateral upward plantar reflexes and decreased pin-prick sensation over the territory of the maxillary nerves.

**Figure 1:**
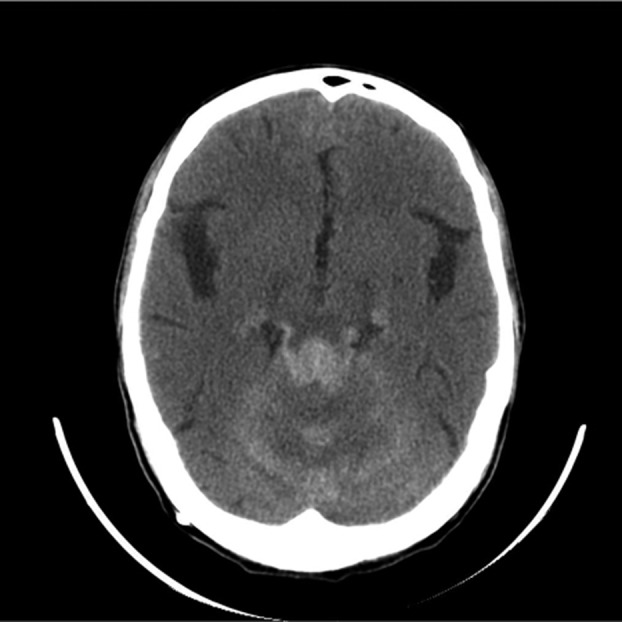
Noncontrast head CT scan is remarkable for hyperattenuated foci in basal meninges and cerebellum.

**Figure 2:**
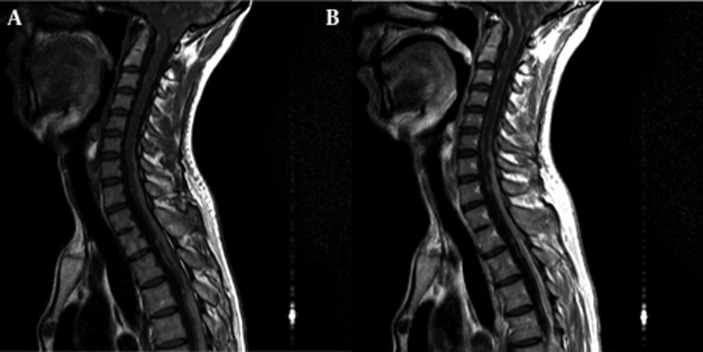
A, Mid-sagittal T1-weighted image through the cervical and upper thoracic region is remarkable for hypersignal leptomeningeal lesions of melanocytoma as a result of T1 shortening effect of their melanin content; B, Mid-sagittal contrast enhanced T1-weighted image is brought in the corresponding level, revealing contrast enhancement in the aforementioned lesions.

**Figure 3:**
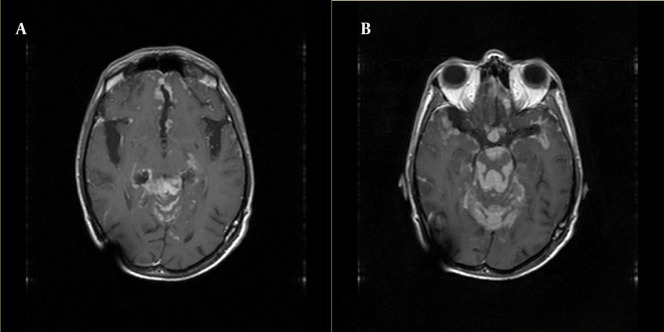
A and B, Axial brain contrast enhanced T1-weighted magnetic resonance images demonstrate nodular and diffuse enhancing regions of leptomeninges throughout basal cisterns and sulci in the interhemispheric fissure, in the location of presumed melanocytoma lesions.

Noncontrast head CT scan showed hyperattenuated foci in both temporal and occipital lobes, basal meninges and the cerebellum (Figure 1). Meanwhile, diffuse leptomeningeal enhancement and multiple well-enhanced nodules along basal cisterns and posterior fossa surface were noted in contrast-enhanced CT scan. Magnetic resonance imaging (MRI) was remarkable for T1 shortening of the leptomeninges and corresponding nodules in precontrast study (Figure 2). Subsequent contrast-enhanced MR imaging of the brain and spine revealed enhancement in the basal cisterns extending throughout the spinal canal (Figure 3). One of the symptomatic intraspinal nodules was resected and surgical specimens consisting of several leptomeningeal- based fragments of soft brown tissue were submitted for pathological study. Gross features were not characteristic for any particular type of meningeal tumor. Light microscopic examination showed the appearance of a neoplastic pigmented lesion. Eventually, immunohistochemical analysis was reactive for S-100 protein, HMB-45 and vimentin, proposing leptomeningeal melanocytoma.

**Figure 4:**
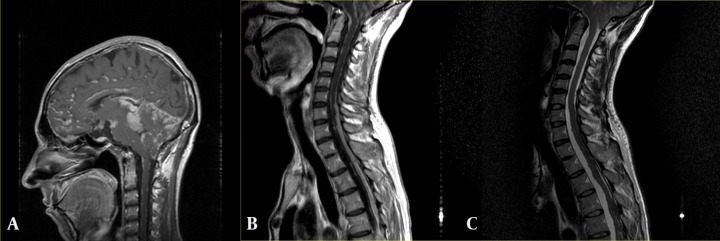
A, Brain mid-sagittal contrast enhanced T1-weighted image clearly depicts leptomeningeal lesions in the posterior fossa and resultant downward tonsillar herniation; B, Similar diffuse enhancing leptomeningeal lesions are notable in contrast enhanced T1 mid sagittal image through the cervical and upper thoracic spine. Tonsillar herniation is also visible in the top of this image. (C) Sagittal T2-weighted image through cervical and upper thoracic spine better delineates tonsillar herniation.

Because of progression of symptoms and development of hydrocephalus, the patient underwent a programmable VP shunt one year later, but thereafter, the patient’s complaints were still present. Further imaging examinations revealed aggravated brain and spinal lesions and a new onset tonsillar herniation (Figure 4). Thus, an elective posterior craniotomy for exploration, microdissection, biopsy taking and partial tumor debulking was undertaken. The patient was discharged with stable and acceptable general condition. Serial follow up visits were planned for the patient; however, unfortunately, corresponding medical records were not available at the time of writing this article.

## 3. Discussion

Primary melanocytic neoplasms are rare lesions which originate from normally existing leptomeningeal melanocytes (3). Melanocytes arise from neural crest elements and are found within the basal layer of the epidermis and the leptomeninges that cover the base of brain and the brain stem (4). Moreover, the highest concentration of melanocytes is seen ventrolateral to the medulla oblongata and upper cervical levels of spinal leptomeniges (5, 6). In general, three main types are considered for these neoplasms, including diffuse melanosis, meningeal melanocytoma and primary malignant melanoma (7). Nowadays, with improvements in neuroimaging and clarification of histological features, meningeal melanocytomas are being diagnosed with increased frequency. Clinical presentation of patients with these tumors typically occurs in their fifth decade. It is seen in women twice as often as men (5). Unlike diffuse melanosis, it is not associated with skin pigmentation. Neurological and clinical features in meningeal melanocytoma is so varied, including the frequent occurrence of hydrocephalus, seizures, chronic basal meningitis, multiple cranial nerve palsies, psychiatric disturbances, intracranial hemorrhage of the meninges or subdural space and myeloradiculopathy. An important consideration is that intracranial and spinal melanocytomas have propensity to arise in proximity to cranial and spinal nerves as they exit the brainstem and spinal cord (7-9).

Biological behavior is variable; incomplete resection may yield in recurrence, an event that may occur because of extensive leptomeningeal involvement or persistence of neglected small foci of tumor. Some believe transition into malignant melanoma does not occur, however, we encountered to four cases of malignant transformation of meningeal melanocytoma, in the literature (10-13). We did not find any description for a case of melanocytoma with tonsillar hernaition; however, we presume that in a case with extensive meningeal involvement, an obstacle for complete debulking may increase the mass effect of the growing lesions consequently leading to this inevitable outcome. Generally, a good postoperative survival rate is expected for patients with meningeal melanocytoma. Hence, the surgeon should be advised of this possible diagnosis in suspected cases of meningioma, especially those involving the posterior fossa or Meckel’s cave, information that makes the surgeon accomplish maximal effort at tumor resection. However, preoperative diagnosis of meningeal melanocytoma is not invariably easy (7). Fortunately, the characteristic shortening of T1 and T2 relaxation times in MR imaging , as a result of the paramagnetic stable free radicals that exist within melanin, may often suggest a diagnosis of a melanocytic leptomeningeal process (7, 11). In addition, there is unique ultra-structural and immunohistochemical characteristics for these varied lesions (7). Another important hint is that the degree of melanization makes the imaging appearance of meningeal melanocytoma variable. Iso- to hyperattenuating lesions with variable contrast enhancement is detected in CT images. While, in MR images, these lesions generally show high signal intensity on T1-weighted images, diminished signal on T2-weighted images and diffuse enhancement after contrast administration (7, 12). Here, an indispensible role is considered for immunohistochemical analysis, a study that can eventually make differentiation of meningeal melanocytoma from other similar pigmented lesions possible. Characteristic immunohistochemical reaction of meningeal melanocytoma is a positive response to antimelanoma (HMB-45), S-100 protein and vimentin (an indicator of cells with mesenchymal origin, which rarely appears in malignant melanoma) antibodies and a negative reaction to epithelial membrane antigen (EMA) (indicator of meningioma) and Leu7 (indicator of schwannoma) (7, 13).

## 4. Conclusion

In appropriate clinical settings, certain radiological findings, especially both T1 and T2 shortening in nodular CNS lesions should propose meningeal melanocytoma in the mind; a primary melanocytic neoplasm in which complete resection heralds a favorable prognosis.
